# Gadolinium-Induced Acute Graft Pancreatitis in a Simultaneous Pancreas-Kidney Transplant Recipient

**DOI:** 10.1155/2022/9533266

**Published:** 2022-08-21

**Authors:** Chiang Sheng Lee, Rachel Yi Ping Tan, Nitesh N. Rao

**Affiliations:** ^1^Renal Unit, Flinders Medical Centre, Flinders Drive, Bedford Park, South Australia, Australia; ^2^College of Medicine and Public Health, Flinders University, Bedford Park, South Australia, Australia; ^3^Renal Unit, Lyell McEwin Hospital, Haydown Road, Elizabeth Vale, Adelaide, South Australia, Australia; ^4^Adelaide Medical School, University of Adelaide, Adelaide, South Australia, Australia

## Abstract

Gadolinium-induced acute pancreatitis is a rare phenomenon associated with the administration of gadolinium-based contrast agents. Only five cases of gadolinium-induced acute pancreatitis have been reported worldwide in patients with native pancreas and none with a pancreatic graft. We present a 32-year-old woman with prior history of simultaneous pancreas-kidney transplant who presented with generalized abdominal pain associated with systemic inflammatory response syndrome requiring admission to the intensive care unit. This occurred within 48 hours after having a magnetic resonance imaging (MRI) with gadolinium for investigation of subacute left optic atrophy. She was noted to have a marked rise in serum lipase, and the computed tomography findings were consistent with acute graft pancreatitis. Other causes of pancreatitis were ruled out, and she was managed conservatively with aggressive hydration, bowel rest, and analgesia with good recovery. This is the first reported case of gadolinium-induced acute graft pancreatitis occurring in a simultaneous pancreas-kidney transplant recipient. Clinicians should consider this rare differential diagnosis as a cause of graft pancreatitis in patients who have received gadolinium-based contrast agents.

## 1. Introduction

Gadolinium-based contrast agents (GBCAs) have been increasingly used in magnetic resonance imaging (MRI) to aid clinicians' diagnostic ability in neurological and inflammatory disorders. It is occasionally used in patients who are not suitable for iodinated contrast agents in computed tomography (CT) imaging [1]. Contrary to the well-described adverse effect of nephrogenic systemic fibrosis caused by GBCAs, little is known about gadolinium-induced acute pancreatitis with only five cases reported worldwide in patients with native pancreas. We describe the first case of gadolinium-induced acute pancreatitis occurring in a simultaneous pancreas-kidney transplant recipient. This is an entity which should be differentiated from transplant graft rejection as the management differs.

## 2. Case Presentation

A 32-year-old woman, who was two years post simultaneous pancreas-kidney (SPK) transplant for type I diabetes, presented to the Emergency Department with sudden onset generalized abdominal pain. The pain was constant and dull in nature. It did not radiate, and there were no exacerbating or relieving factors. It was associated with fever, nausea, and vomiting, but there was no alteration in bowel habits leading up to this sudden abdominal pain. She denied any recent trauma, abnormal bowel movements, or urinary symptoms. She denied any illicit drug or alcohol use. The only relevant history was a recent magnetic resonance imaging (MRI) brain with gadolinium-based contrast agent (GBCA), gadobutrol (*Gadovist*), 48 hours prior to investigate for subacute left optic atrophy.

As for her SPK transplant, it was a 3/6 human leukocyte antigen mismatch with positive angiotensin II receptor type 1 antibody of 33.7 IU/ml. Her baseline renal graft function was 85 *μ*mol/L. There was no history of pancreatic graft rejection or acute pancreatitis.

Her other past medical history included migraine, recurrent urinary tract infections, and recent investigation for left vision loss. Regular medications included mycophenolate mofetil 750 mg twice daily, tacrolimus 3 mg twice daily, prednisolone 10 mg daily, pantoprazole 40 mg daily, trimethoprim-sulfamethoxazole 160/800 mg twice weekly, candesartan 8 mg daily, atorvastatin 20 mg daily, aspirin 100 mg daily, and amitriptyline 25 mg daily.

On arrival to the Emergency Department, she was observed to be in shock with cool peripheries and recorded blood pressure of 90/29 mmHg. She was tachycardic at 100 beats per minute and hypothermic at 34.3°C. The blood glucose level was 6.5 mmol/L. Examination was remarkable for abdominal tenderness over the pancreatic graft. Renal graft was non-tender and there was no bruit audible.

Initial arterial blood gas demonstrated lactate acidosis with pH of 7.24, pO_2_ of 106 mmHg, pCO_2_ of 44 mmHg, HCO_3_ of 18 mmol/L, and lactate of 3.2 mmol/L. Urine analysis showed trace of ketones. Urinary beta-human chorionic gonadotropin was negative. Initial investigations demonstrated white cell count of 22.34 × 10^9^/L, haemoglobin of 141 g/L, platelet count of 409 × 10^9^/L, acute renal injury with serum creatinine of 159 *μ*mol/L (baseline of 85 umol/L), urea of 12.4 mmol/L, sodium of 145 mmol/L, and potassium of 4.6 mmol/L. Blood glucose was 8.4 mmol/L. Serum lipase was elevated at 3368 U/L (normal range: 0–60 U/L). Liver function test was unremarkable, and C-reactive protein was not elevated. 12-hour tacrolimus trough level was 6.2 ug/L (Table 1). Donor specific antibody was negative.

**Figure 1:**
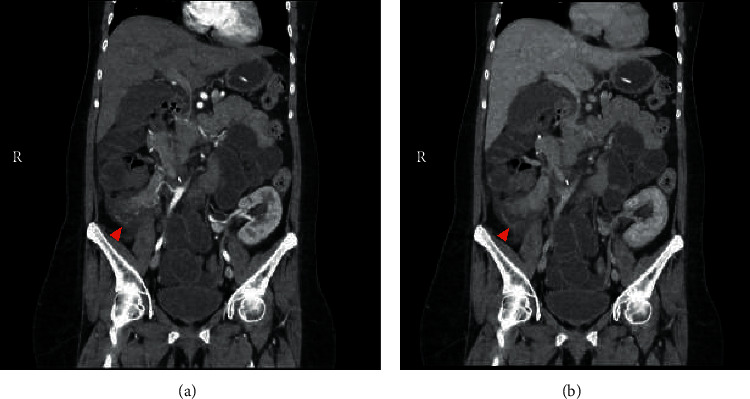
Computed tomography of the abdomen in coronal view. (a) Arterial phase demonstrates satisfactory enhancement of pancreatic artery (red arrow). (b) Venous phase demonstrates poor enhancement of pancreatic tail with surrounding inflammatory changes (red arrow).

Resuscitation was undertaken with intravenous fluids, and antibiotics for presumed intraabdominal sepsis were initiated. Computed tomography (CT) of her abdomen demonstrated inflammatory changes of transplanted pancreatic graft with poor enhancement suggestive of graft pancreatitis (Figure 1). There was an absence of bladder distension, hydroureter, or hydronephrosis of the transplanted kidney graft. Cytomegalovirus polymerase chain reaction (PCR) was negative. Blood and urine cultures were unremarkable, and antibiotics were discontinued within 48 hours. Pancreatic graft rejection was considered less probable given the improvement in clinical condition, normal fasting C-peptide level of 915 pmol/L, normal blood glucose levels, and therapeutic drug levels (Table 1). After excluding other causes of allograft pancreatitis, she was treated for gadolinium-induced acute pancreatitis. She improved with conservative management and was discharged after 3 days. She has had no further episodes of acute pancreatitis upon follow-up for 12 months.

## 3. Discussion

Gadolinium-induced acute pancreatitis (gadolinium-induced AP) is a rare phenomenon. Despite the safety and tolerability of GBCAs, there are five cases which reported gadolinium-induced AP in patients with native pancreases [2–6]. It has been described to develop between three to six hours after administration of GBCAs including gadodiamide (Omniscan) [2, 6], gadolinium-diethylenetriamine pentaacetic acid (Gd-DTPA) [4], gadobenate dimeglumine (MultiHance) [3], and gadoterate meglumine [5].

Multiple mechanisms of GBCA toxicity have been proposed. In a recent review [7], they suggest the main factor for GBCA toxicity is the dissociation of gadolinium ion (Gd^3+^) from chelated complexes leading to non-chelated Gd^3+^ being retained in organ tissues. Other studies also proposed that free Gd^3+^ leads to cytokine stimulation, macrophagic activation, and attraction of fibroblasts. However, none of the animal studies recorded the effects of GBCA on the pancreas.

There are no formalized criteria for gadolinium-induced AP. It is a clinical diagnosis that correlates with the development of acute pancreatitis following the administration of gadolinium. The diagnosis of acute pancreatitis itself is based on the revised Atlanta Classification which requires at least 2 of the 3 characteristics: abdominal pain consistent with pancreatitis, serum lipase value 3 times the upper limit of normal, and supportive imaging findings [5]. CT findings suggestive of acute pancreatitis include pancreatic enhancement with increased attenuation in peripancreatic fat, peripancreatic fluid collection, or pancreatic necrosis in severe cases [8]. Our patient fulfilled all criteria. She was low risk for severe pancreatitis based on the modified Glasgow system [9] (Table 1).

There are three main differences compared to the other cases reported. Firstly, this is the first case of acute pancreatitis due to gadobutrol occurring in a pancreatic graft. She also developed symptoms after 48 hours of GBCA administration. Given the temporal relationship of GBCA administration and development of graft pancreatitis with no further recurrence during subsequent follow-ups while being on the same immunosuppressants, drug-induced pancreatitis attributed to her regular medications was deemed less likely as per the Mallory and Kern criteria [10].

Another challenging aspect for our patient was to differentiate between other causes of transplant graft pancreatitis including pancreatic graft rejection. Transplant graft pancreatitis is the second most common complication after vascular thrombosis [11]. Reported risk factors for graft pancreatitis include exocrine bladder drainage, mechanical stricture, direct mechanical pressure to the graft, intraparenchymal microvascular thrombosis, recurrent infection surrounding graft, occlusion of Oddi's sphincter due to rejection, and cytomegalovirus infection [11].

Similarly, pancreatic graft rejection can manifest as acute pancreatitis with elevated serum lipase with or without accompanying graft tenderness as the graft itself is insensate [12]. The evaluation of pancreatic graft rejection should be accompanied by other features such as elevated glycated haemoglobin, hyperglycaemia, low fasting C-peptide, and exclusion of other structural pancreatic pathology by imaging. A core needle biopsy of the graft pancreas is the gold standard for diagnosis and should be considered if graft rejection is suspected. This will distinguish between antibody mediated rejection from acute cellular rejection based on the Banff classification. The presence of DSA can also aid in the diagnosis of graft rejection as it is an independent predictor of graft failure [12]. In our case, pancreatic graft biopsy was not undertaken as there was rapid improvement in her clinical parameters in addition to having normal blood glucose and C-peptide levels and negative DSAs.

While both entities can lead to elevated amylase or lipase levels, graft pancreatitis would have a significantly higher reading compared to pancreatic graft rejection. Imaging adjacent to pancreatic allograft showing pancreatic infiltration, oedema, and peripancreatic fluid would also favour graft pancreatitis [11].

To our knowledge, this is the first case report of gadolinium-induced acute graft pancreatitis in a patient with simultaneous pancreas-kidney transplant. While this is a rare occurrence, physicians should consider gadolinium-induced pancreatitis as a differential diagnosis for pancreatic graft pancreatitis.

## Figures and Tables

**Table 1:** Relevant laboratory parameters during the patient's hospital admission (the 8 criteria included in the modified Glasgow system to predict the severity of acute pancreatitis at 48 hours include age, white cell count, partial pressure of oxygen, serum calcium, urea, lactate dehydrogenase, serum albumin, and blood glucose [9]).

Test	3 months prior	Day 0	Day 1	Day 2	Day 3	Normal Range
Haemoglobin (g/L)	146	141	123	116	122	115–155
White cell count (×10^9^/L)	8.98	22.34	15.66	13.73	13.95	4–11
Absolute neutrophil count (×10^9^/L)	6.70	12.96	14.50	10.40	11.20	1.8–7.5
Platelet count (×10^9^/L)	304	409	285	252	250	150–450
C-Reactive protein (mg/L)		12.4	—	98.8	—	0–8
Urea (mmol/L)	8.6	12.4	10.8	7.6	4.4	2.7–8.0
Creatinine (umol/L)	97	159	115	108	86	45–90
Serum calcium (mmol/L)	—	2.33	2.21	2.02	2.11	2.10–2.60
Blood glucose (mmol/L)		8.4	7.2	4.8	4.7	3.2–5.5
Lipase (U/L)	20	3368	541	115	30	0–60
C-Peptide (pmol/L)		915	—	—	—	166–1466
Lactate dehydrogenase (U/L)		249	418	414	382	120–250
Alanine aminotransferase (U/L)		31	38	32	20	0–55
Total cholesterol (mmol/L)		3.4	—	—	—	
Triglyceride (mmol/L)		0.5	—	—	—	
Tacrolimus level (ug/L)		5.0	—	—	6.2	5–15

## Data Availability

No data were used to support this study.
